# Decreased SIRT3 in aged human mesenchymal stromal/stem cells increases cellular susceptibility to oxidative stress

**DOI:** 10.1111/jcmm.12395

**Published:** 2014-09-11

**Authors:** Xue-Qing Wang, Yong Shao, Chong-Yi Ma, Wei Chen, Lu Sun, Wei Liu, Dong-Yang Zhang, Bi-Cheng Fu, Kai-Yu Liu, Zhi-Bo Jia, Bao-Dong Xie, Shu-Lin Jiang, Ren-Ke Li, Hai Tian

**Affiliations:** a Department of Cardiovascular Surgery, Second Affiliated Hospital of Harbin Medical UniversityHarbin, China; b Key Laboratories of Myocardial Ischemia Mechanism and Treatment, Harbin Medical University, Ministry of EducationHarbin, China; c Toronto General Research Institute, University Health Network and Department of Surgery, Division of Cardiac Surgery, University of TorontoToronto, ON, Canada

**Keywords:** SIRT3, oxidative stress, ageing, mesenchymal stromal/stem cells, myocardium, antioxidants

## Abstract

Sirtuin3 (SIRT3) is an important member of the sirtuin family of protein deacetylases that is localized to mitochondria and linked to lifespan extension in organisms ranging from yeast to humans. As aged cells have less regenerative capacity and are more susceptible to oxidative stress, we investigated the effect of ageing on SIRT3 levels and its correlation with antioxidant enzyme activities. Here, we show that severe oxidative stress reduces SIRT3 levels in young human mesenchymal stromal/stem cells (hMSCs). Overexpression of SIRT3 improved hMSCs resistance to the detrimental effects of oxidative stress. By activating manganese superoxide dismutase (MnSOD) and catalase (CAT), SIRT3 protects hMSCs from apoptosis under stress. SIRT3 expression, levels of MnSOD and CAT, as well as cell survival showed little difference in old *versus* young hMSCs under normal growth conditions, whereas older cells had a significantly reduced capacity to withstand oxidative stress compared to their younger counterparts. Expression of the short 28 kD SIRT3 isoform was higher, while the long 44 kD isoform expression was lower in young myocardial tissues compared with older ones. These results suggest that the active short isoform of SIRT3 protects hMSCs from oxidative injury by increasing the expression and activity of antioxidant enzymes. The expression of this short isoform decreases in cardiac tissue during ageing, leading to a reduced capacity for the heart to withstand oxidative stress.

## Introduction

Epidemiological data illustrate that the global population is ageing. Many clinical abnormalities with higher morbidity and mortality are linked with an ageing population. Although many factors contribute to diseases in populations as they grow older, ageing is a pivotal independent factor that restricts the capacity of repair and regeneration in the elderly 1. In aged patients, bone marrow stem cells have decreased tissue repair capacity. Our previous research has revealed the tight association between ageing and cell function, indicating that cell proliferative capacity, biological activity and the ability to adapt to injury decreases in old human mesenchymal stromal/stem cells (hMSCs) 2. Furthermore, genetic modification of old hMSCs with tissue inhibitor of matrix metalloproteinase-3 (TIMP-3) or VEGF improves the tissue repair function of aged cells and in some cases renders them indistinguishable from young cells 3. Therefore, rejuvenation of ageing cells is a plausible method for improving tissue repair in aged patients.

Oxidative stress is a driving factor behind a series of detrimental cell events that includes ageing, apoptosis and tumour growth 4–7. The mitochondria are the main compartments of respiration and metabolism in eukaryotic cells and are thus enriched in high energy-consuming organs of the body and are critical in the defence against oxidative stress 8. Maintaining the normal function of mitochondria is essential to ensure the efficient scavenging of reactive oxygen species (ROS) and prevent cell senescence and other stress-related events that can be induced by increased concentrations of ROS 9–11.

Sirtuin3 (SIRT3) belongs to the highly conserved Sirtuin family, which contains seven members in humans that are all homologous to silent information regulator 2 (Sir2) in yeast 11–14. It is known that SIRT3 localizes to mitochondria and is likely linked to extended lifespan of humans 15–17. Human SIRT3 has two isoforms: a full-length isoform (fl-SIRT3, 44 kD) and a short one (sh-SIRT3, 28 kD) 11,15,18–20. The short isoform is generated by cleaving a 142 residue N-terminal mitochondrial localization sequence from the long form. This cleavage event occurs in the mitochondria 11,12. Although which isoform is the active form remains controversial, most studies in human and mammalian cells suggested that the shorter, 28 kD isoform is the functional one 11,15,18–20. By participating in mitochondrial metabolism, SIRT3 counteracts oxidative stress, defends cells against apoptosis and prevents cell ageing and tumour formation 11,12,15–17,21,22. In myocardial tissues, SIRT3 reduces levels of ROS by deacetylating the transcription factor Foxo3a (forkhead box O3a), which can then enter the nucleus and bind to the promoter of the genes encoding manganese superoxide dismutase (MnSOD) and catalase (CAT) 21,23,24. This process increases MnSOD and CAT gene and protein expression and function. Other reports also demonstrate that SIRT3 increases the activity of these antioxidant enzymes by the nuclear factor κB (NF-κB pathway) 25. Elevated MnSOD and CAT can protect tissue from injury induced by ROS 26–28.

Though there is significant evidence to support a critical role for SIRT3 in human longevity, the majority of previous studies are based on gene polymorphisms in humans and do not examine SIRT3 function at the molecular level 29–33. Here, we compared the SIRT3 expression in young and old hMSCs and myocardial tissues with and without oxidative stress to elucidate a potential relationship between human SIRT3 and ageing as well as a role for this protein in MnSOD and CAT function. The correlation between SIRT3 expression, oxidative stress and senescence implies that manipulation of SIRT3 levels could lead to more effective therapeutics in aged populations.

## Materials and methods

### Human bone marrow and myocardial tissue collection

Both bone marrow and myocardial tissue donors provided their written informed consent to participate in this study, while the rights of infants and young children were entrusted to their parents or other direct relatives. All human protocols were reviewed and approved by the Ethics Committee of Harbin Medical University and conformed to World Medical Association guidelines.

Human bone marrow was acquired from the sternum of patients who underwent cardiac surgery at the Second Affiliated Hospital of Harbin Medical University. Young hMSCs samples came from six different patients of 0–9 years (mean = 4.7 ± 3.2, *n* = 6), and old hMSCs were from patients 57–66 years of age (mean = 61.5 ± 3.4, *n* = 6).

Human myocardial tissue was obtained from patients that required atrial volume reduction during cardiac surgery. All tissues were conserved in liquid nitrogen with a frozen pipe (Corning, Corning, New York, USA). The young myocardial tissue was obtained from donors aged 0–10 years old (mean = 5.1 ± 3.7, *n* = 8), while the old myocardial tissues were obtained from patients aged 54–68 years (mean = 58.8 ± 5.2, *n* = 8).

### hMSC isolation and culture

Human mesenchymal stromal/stem cells were isolated with a Ficoll-Paque gradient (1.073 g/ml density; GE Healthcare, Little Chalfont, Buckinghamshire, UK) by centrifugation at 1330 × g for 20 min. The mononuclear cells were collected and rinsed twice, and then plated into 25 cm^2^ culture flasks (Corning) in Iscove’s modified Dulbecco medium (Life Technologies, Carlsbad, California, USA) supplemented with 10% foetal bovine serum (Biological Industries, Kibbutz Beit Haemek, Israel). Finally, the cells were incubated at 37°C with 5% CO_2_. Third generation hMSCs were harvested for the following experiments.

### Transfection of SIRT3 into hMSCs

A plasmid containing a SIRT3 expression gene (pSIRT3) was constructed by GENEPHARMA on a pEX-1 (pGCMV/MCS/EGFP/NEO) backbone and amplified and prepared using the TIANprep mini plasmid kit (TIANGEN, Beijing, China). Transfection was done in accordance with the manufacturer’s instructions (X-tremeGENE HP DNA transfection reagent, Roche, Penzberg, Upper Bavaria, Germany). The ratio of plasmid DNA (μg) to transfection reagent (μl) was 1:3. After 72 hrs culture, transfection efficiency was assayed by fluorescence-activated cell sorting (FACS; BD, Franklin Lakes, New Jersey, USA).

### hMSCs damage measurement

A total of eight different experimental groups were used in this study, six using young hMSCs and two using old cells. For young cells, the experimental groups were: control (no treatment), hydrogen peroxide (H_2_O_2_) only, pEX-1 (empty vector pEX-1 transfection), pEX-1+ H_2_O_2_, pSIRT3 transfection and pSIRT3+ H_2_O_2_. The experimental groups for old cells were control and H_2_O_2_-treated. Transfection was performed when cells reached 70–90% confluence. After transfection or mock transfection, cells were treated with H_2_O_2_ (1 mM) for 1 hr. All cells were then harvested for the following experiments.

### Gene expression measurement

RNA was extracted directly from hMSCs using TRIzol Reagent (Life Technologies, Carlsbad, California, USA) in 6-well plates. Myocardial tissues were powdered and their RNA was extracted. RNA samples were treated with DNase 1 (Sigma-Aldrich, St. Louis, Missouri, USA) according to the manufacturer’s protocol. Reverse transcription was performed with AccuPower RocketScript RT PreMix (Bioneer, Alameda, California, USA) according to the suggested protocol. Gene expression was determined by real-time PCR with AccuPower 2× Greenstar qPCR Master Mix (Bioneer) in a thermal cycler (Bio-Rad, Hercules, California, USA). The primers were designed and synthesized by Bioneer Corporation as follows: SIRT3 forward, 5′-GCATCCCTGCCTCAAAGC-3′, SIRT3 reverse, 5′-CGTCAGCCCGAATGTCCTC-3′; CAT forward, 5′-TGCTGAGGTTGAACAGATAG-3′, CAT reverse, 5′-CCGTCACGCTGGTAGTT-3′; MnSOD forward, 5′-TGGTGGTCATATCAATCATAGC-3′, MnSOD reverse, 5′-ATTTGTAAGTGTCCCCGTTC-3′; β-actin forward, 5′-CCCAGCACAATGAAGATCAAGATCAT-3′, β-actin reverse, 5′-ATCTGCTGGAAGGTGTACAGCGA-3′.

### Protein level measurement

Protein expression levels of SIRT3 and MnSOD were determined by Western blot. Protein was extracted from cells and myocardial tissues using a RIPA lysis buffer (Solarbio, Beijing, China) with a protease inhibitor cocktail tablet (cOmplete ULTRA Mini EDTA-free, Easypack, Roche). Protein density of all samples was measured with a BCA Protein Assay Kit (Beyotime, Haimen, Jiangsu, China) according to the manufacturer’s protocol on a microplate reader (TECAN, Männedorf, Switzerland). Antibodies used were as follows: SirT3 (C73E3) rabbit mAb (Cell Signaling Technology, Danvers, Massachusetts, USA), rabbit monoclonal antibody against SOD2 (MnSOD, OriGene, Beijing, China), mouse anti-β-actin monoclonal antibody (ZSGB-BIO), fluorescein-conjugated affinipure goat anti-rabbit IgG (ZSGB-BIO) and fluorescein-conjugated affinipure goat antimouse IgG. CAT protein level was tested with a human CAT ELISA kit (BG, Shanghai, China). For ELISA, cultured cells were collected in phosphate buffered saline and sonicated, while myocardial tissues were ground on ice and proteins were extracted.

### Enzyme activity measurement of CAT and MnSOD

For tests of enzyme activity, cultured cells and myocardial tissue samples were collected and the protein extracted as outlined above. Enzyme activity was detected using the Catalase Assay and MnSOD Assay kits (Beyotime).

### Cell survival and apoptosis evaluation

Cell Counting Kit-8 (CCK8; Dojindo, Kumamoto, Japan) was used to determine cell survival according to the manufacturer’s instructions. Cells were seeded into 96-well plates at a density of 10,000 cells/well (3 wells/group). Samples were then detected in a microplate reader after 2 hrs in culture. Cell apoptosis was determined by a PE Annexin V Apoptosis Detection kit I (BD, San Diego, California, USA) and 4′,6-diamidino-2-phenylindole (DAPI, Sigma-Aldrich) staining. FACS and cell counting were used to assay and analyse the rate of apoptosis.

### Statistical analysis

All data are expressed as mean ± SD. Comparison between two groups was done by a two-tailed Student’s *t*-test. One-way anova was used to determine the significance between three or more experimental groups. *P* < 0.05 was determined statistically significant.

## Results

### SIRT3, CAT and MnSOD levels in young hMSCs decrease under oxidative stress, correlated with decreased cell survival and increased apoptosis

**Figure 1:**
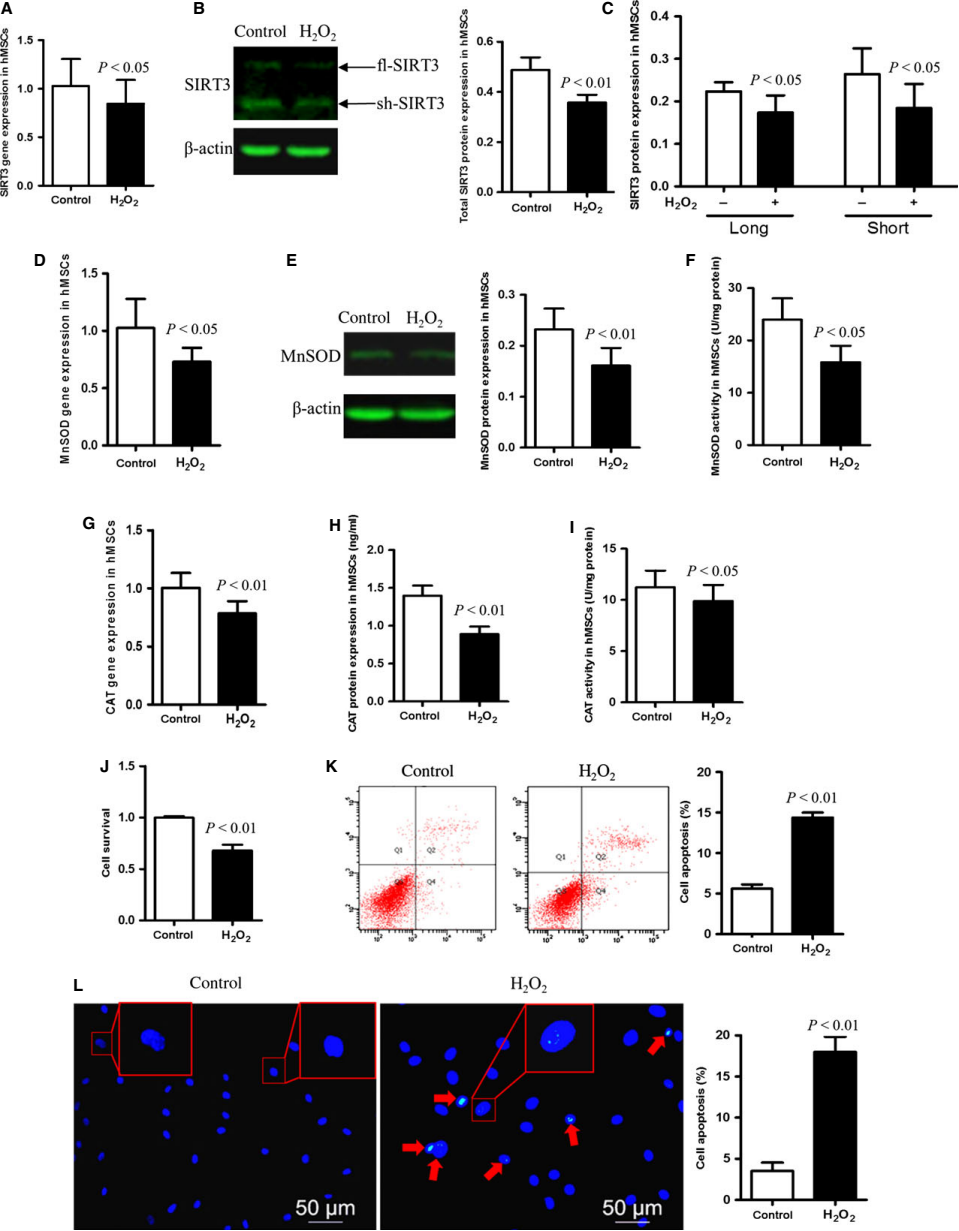
Oxidative stress leads to decreased SIRT3, CAT and MnSOD levels in young hMSCs correlated with decreased cell survival and increased apoptosis. (**A**) hMSCs was treated with 1 mM H_2_O_2_ for 1 hr and gene expression was evaluated by real-time PCR. SIRT3 mRNA expression was reduced in young hMSCs under oxidative stress. (**B** and **C**) show total SIRT3 protein expression levels as well as the expression levels of its two isoforms (full-length SIRT3, fl-SIRT3 and short SIRT3, sh-SIRT3) before and after H_2_O_2_ treatment, assessed by Western blot with β-actin serving as an internal control. (**D** and **G**) show MnSOD and CAT gene expression before and after treatment. (**E** and **H**) MnSOD and CAT protein expression assayed by Western blot. (**F** and **I**) Enzymatic activity of MnSOD and CAT in control and H_2_O_2_-treated groups. (**J**) Cell survival evaluated by Cell Counting Kit-8 (CCK8). (**K** and **L**) Cell apoptosis assessed by flow cytometry and 4′,6-diamidino-2-phenylindole (DAPI) staining in control and H_2_O_2_-treated groups. Red arrows indicate apoptotic cells and some cells are enlarged to show the stages of apoptotic degradation: the nuclear membrane edge of phase I apoptotic cells is rippled (left panel, insets), while the chromatin of phase II apoptotic is highly condensed and marginalized (right panel, red arrows and inset), and finally apoptotic bodies are formed.

To investigate the association of SIRT3 and oxidative stress in human cells, hMSCs were exposed to H_2_O_2_. Compared with controls cells, both mRNA and total SIRT3 protein (t-SIRT3) levels decreased after H_2_O_2_ treatment (Fig. 1A and B). We found that SIRT3 exists in two isoforms, the longer fl-SIRT3 (44 kD) and a shorter sh-SIRT3 (28 kD, Fig. 1C). Both fl-SIRT3 and sh-SIRT3 are reduced after H_2_O_2_ treatment, but there was no difference between fl-SIRT3 and sh-SIRT3 levels either before or after incubation with H_2_O_2_ (Fig. 1C). mRNA levels of MnSOD and CAT in the cultured cells declined after oxidative injury (Fig. 1D). The protein levels of these antioxidant enzymes and their enzymatic activity also decreased after oxidative stimuli (Fig. 1E–I). Cell survival was evaluated by CCK8, while cell apoptosis was assayed by FC and cell counting, and we found that oxidative injury decreased cell survival rate and increased cell apoptosis significantly in H_2_O_2_-treated cells (Fig. 1J–L).

### SIRT3 is a stress-responsive protein, and overexpression of SIRT3 increases the expression of its downstream targets

**Figure 2:**
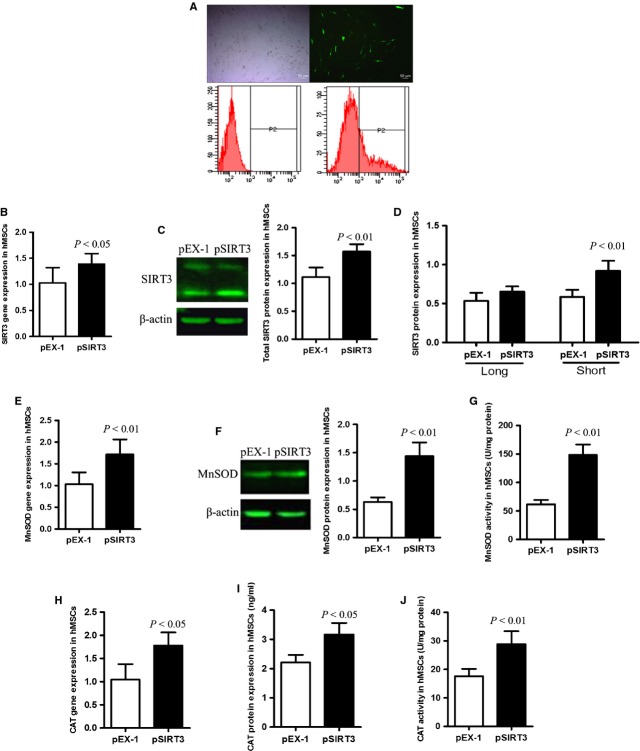
Transfection of pSIRT3 enhances SIRT3, CAT and MnSOD expression in young hMSCs. (**A**) Transfection efficiency of young hMSCs (23.6% ± 3.1%, *n* = 3) was detected by fluorescence-activated cell sorting. (**B** and **C**) showed SIRT3 gene and protein expression in the pEX-1- and pSIRT3-transfected groups. (**D**) It shows full-length (fl-SIRT3) and short SIRT3 (sh-SIRT3) expression levels in the pEX-1- and pSIRT3-transfected groups. (**E**–**I**) Gene and protein levels of MnSOD and CAT were increased after pSIRT3 transfection compared with the pEX-1-transfected group. (**G** and **J**) Enzymatic activity of MnSOD and CAT were also enhanced after pSIRT3 transfection compared with the pEX-1-transfected group.

To study the function of SIRT3 in hMSCs, we overexpressed SIRT3 in the cultured cells. FC data showed that transfection efficiency was 23.6% ± 3.1% (Fig. 2A). pSIRT3 transfection enhanced gene and total protein expression of SIRT3 significantly in comparison with the empty plasmid group (Fig. 2B and C). Interestingly, only sh-SIRT3 expression significantly increased after pSIRT3 transfection compared with empty plasmid transfection (Fig. 2D). We further observed that the increased gene and protein expression as well as enzymatic activity of MnSOD and CAT were associated with increased SIRT3 expression (Fig. 2E–J). As the dominant isoform expression of SIRT3 after pSIRT3 transfection is the sh-SIRT3 isoform, we hypothesize that expression of sh-SIRT3 is more correlated with the increased expression of MnSOD and CAT than is fl-SIRT3 expression, indicating the potential intimate association between sh-SIRT3 and these antioxidant enzymes and their activities.

### SIRT3 overexpression elevates antioxidant levels and cell survival of young hMSCs

**Figure 3:**
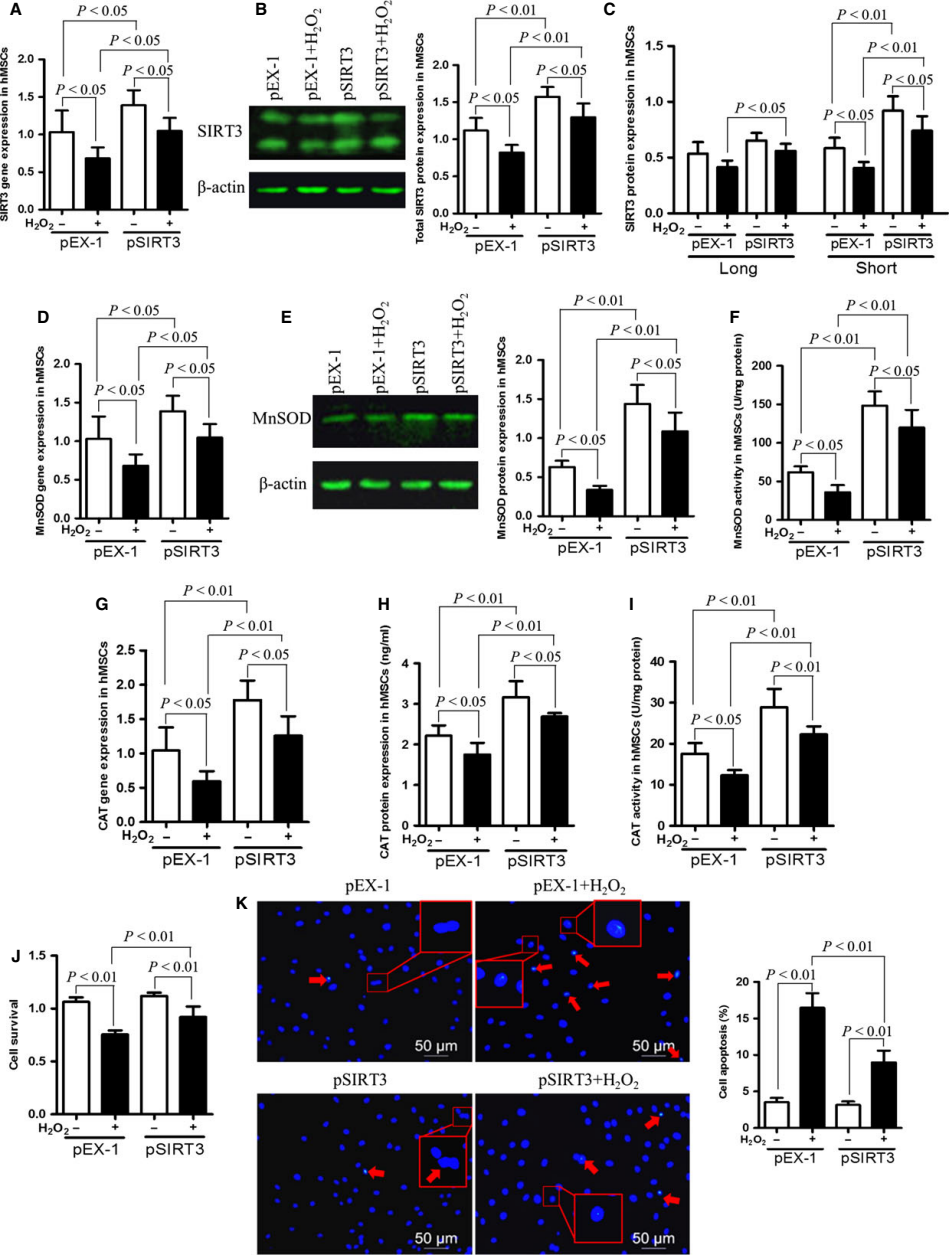
SIRT3 overexpression enhances the ability of young hMSCs to protect against oxidative stress. (**A** and **B**) SIRT3 gene and protein expression in pEX1 and pSIRT3 transfection groups with and without H_2_O_2_ treatment. (**C**) Full-length and sh-SIRT3 expression in pEX-1- and pSIRT3-transfected cells with or without oxidative stress. (**D**–**H**) Gene and protein levels of MnSOD and CAT are higher in the pSIRT3 group than the pEX1 group with and without H_2_O_2_ treatment. (**F** and **I**) The activity of MnSOD and CAT in pEX-1 and pSIRT3 groups with and without H_2_O_2_ treatment. (**J** and **K**) Cell survival and cell apoptosis in pEX-1 and pSIRT3 groups with or without H_2_O_2_ treatment. Overall, MnSOD and CAT expressions, function and cell survival are increased, while cell death is decreased in cells transfected with pSIRT3 than those transfected with pEX-1. Oxidative stress reduces these effects, but cells transfected with the SIRT3 expression construct are still protected against H_2_O_2_-induced cell death and show increased antioxidant expression and function in the presence of H_2_O_2_ than cells expressing empty plasmid.

Given that SIRT3 has been found to protect cells and organs from oxidative stress, we treated young hMSCs transfected with pEX-1 and pSIRT3 with H_2_O_2_ and assayed SIRT3 expression and antioxidant enzyme levels. We found that SIRT3 levels were significantly greater in the group transfected with SIRT3 gene than that in the plasmid control group with or without H_2_O_2_ treatment (Fig. 3A and B). Of the two forms of SIRT3, fl-SIRT3 expressions were similar between SIRT3 transfected and empty plasmid control groups. Also, no difference was seen in fl-SIRT3 expression levels before and after H_2_O_2_ treatment in transfected cells. However, fl-SIRT3 expression was higher in the pSIRT3 group than the pEX-1 group under the same levels of oxidative stress. Moreover, pSIRT3 transfection increased sh-SIRT3 level significantly. Although sh-SIRT3 expression decreased after treatment with H_2_O_2_ in both the pEX-1 and pSIRT3 groups, expression of sh-SIRT3 was still higher in the pSIRT3+ H_2_O_2_ cells compared to the pEX-1+ H_2_O_2_ group (Fig. 3C). Measurement of MnSOD and CAT mRNA, protein and enzymatic activity levels showed they decreased significantly after H_2_O_2_ treatment, whereas SIRT3 gene transfection increased MnSOD and CAT levels compared with the empty vector control group (Fig. 3D–I). In addition, the elevated antioxidant enzyme activities in the SIRT3 gene-transfected group was associated with improved cell survival after H_2_O_2_ treatment. Figure 3J illustrates that the survival rate of pSIRT3-transfected cells treated with H_2_O_2_ was higher than that in the pEX-1+ H_2_O_2_ group. Apoptosis of transfected cells was markedly increased after H_2_O_2_ treatment. However, the percentage of cell apoptosis in pSIRT3-transfected cells with H_2_O_2_ was significantly reduced compared to pEX-1+ H_2_O_2_ (Fig. 3K). Therefore, we suggest that SIRT3 overexpression enhances antioxidant activity and cell survival of hMSCs, and this function is likely achieved by enhancing CAT and MnSOD expression.

### Effect of age on SIRT3, MnSOD and CAT levels of hMSCs and myocardial tissues under normal conditions

**Figure 4:**
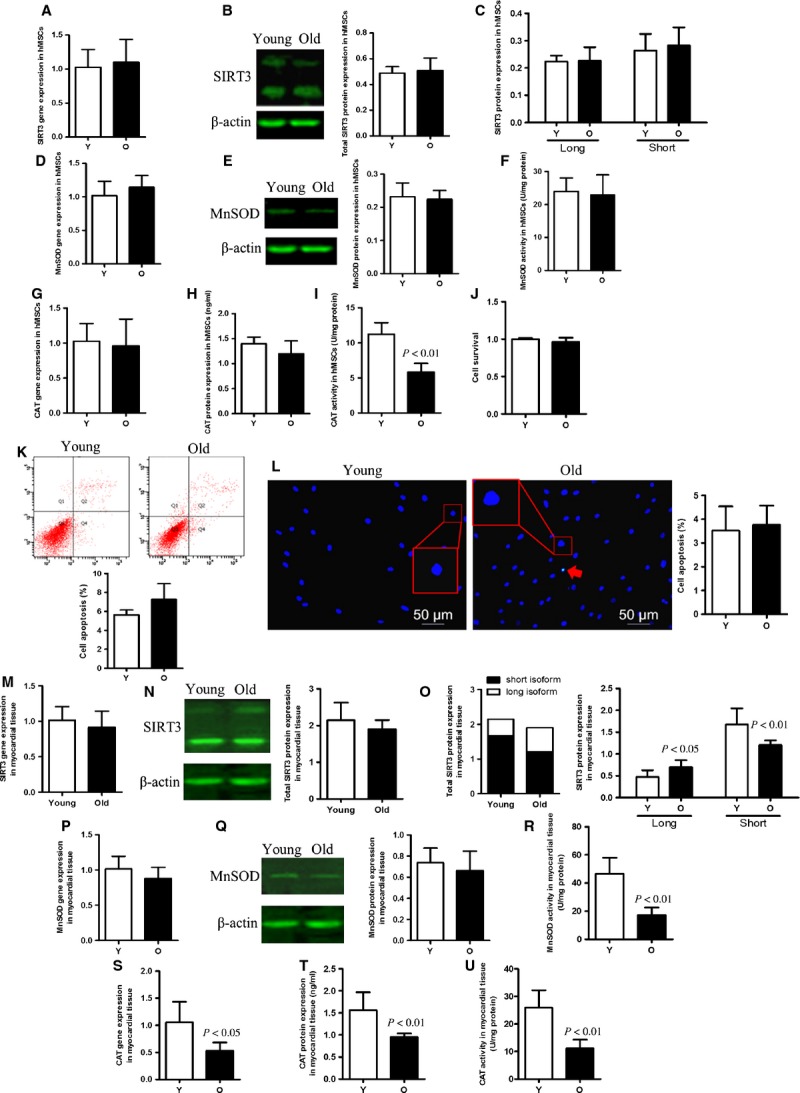
SIRT3, MnSOD and CAT expression in young and old hMSCs and myocardial tissues. (**A** and **B**) There were no differences in SIRT3 gene and protein expression between young (Y) and old (O) hMSCs in normal culture conditions. (**C**) The protein expression of full-length (fl-SIRT3) and the short isoform of SIRT3 (sh-SIRT3) in young and old hMSCs. (**D**–**H**) There were no differences in MnSOD and CAT gene and protein expression between young and old hMSCs. (**F** and **I**) The enzymatic activity of MnSOD and CAT in the young and old cell groups. (**J**–**L**) Cell survival and apoptosis in young cells compared with old cells. No differences in cell survival or apoptosis were seen in the absence of cellular stress. (**M** and **N**) mRNA and total protein expression of SIRT3 in young and old myocardial tissues. (**O**) fl-SIRT3 and sh-SIRT3 expression in young and old myocardial tissues. Despite there being no difference in SIRT3 gene expression, sh-SIRT3 is found at a significantly higher level in young compared to old cells, while fl-SIRT3 is decreased in these cells. (**P**–**T**) MnSOD and CAT gene and protein levels in young and old cell groups. (**R** and **U**) Both MnSOD and CAT activities are decreased in old myocardial tissue.

To examine the role of SIRT3 in ageing of human cells and organs, hMSCs were harvested and cultured, and cardiac tissues were collected from young and aged donors. Gene expression and protein levels of SIRT3, both long and short isoforms, were similar in the cells from young and old donors (Fig. 4A–C). Similarly, ageing did not change gene expression and protein levels of either antioxidant enzyme MnSOD or CAT (Fig. 4D, E, G and H). There was also no difference between young and aged groups in MnSOD activity, but CAT activity in aged cells was significantly lower than that in young cells (Fig. 4F and I). Also, little disparity was observed in terms of cell survival and apoptosis between young and old groups under normal conditions (Fig. 4J–L). Likewise, we found no significant difference in SIRT3 mRNA or total SIRT3 protein expression of myocardial tissues between young and old patient groups (Fig. 4M and N). However, sh-SIRT3 protein expression in young tissue was significantly higher than that found in old tissue (Fig. 4O). In contrast, fl-SIRT3 expression was markedly increased in the aged group. In both young and old tissue samples, the majority of total SIRT3 protein present in myocardial tissues was the shorter sh-SIRT3 isoform. CAT mRNA and protein levels were markedly higher in the young group than in the old group (Fig. 4S and T), whereas little difference was seen in MnSOD expression levels (Fig. 4P and Q). Similarly, significantly higher enzymatic activity of CAT and MnSOD was observed in young rather than old myocardial tissues (Fig. 4R and U).

### Comparison of SIRT3 levels and cellular susceptibility of young and old hMSCs to oxidative stress

**Figure 5:**
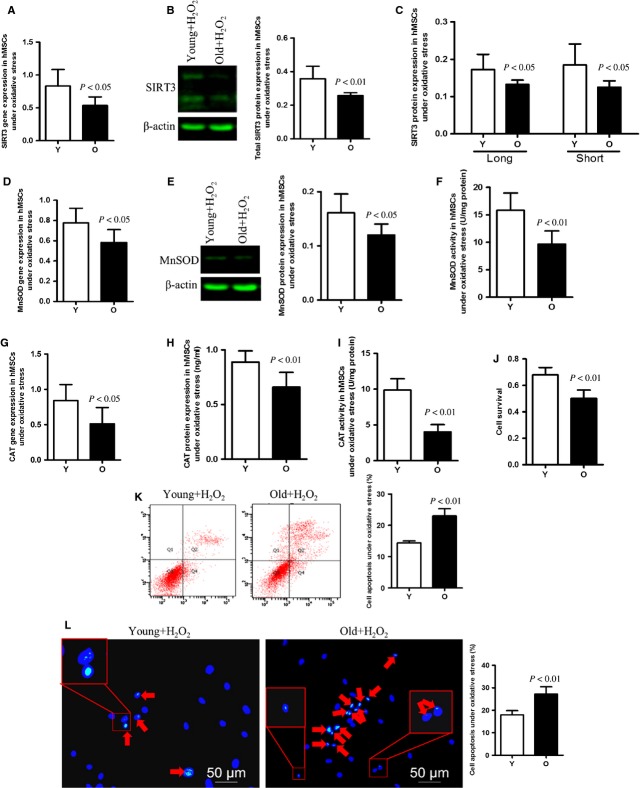
The antioxidant ability of old hMSCs was significantly decreased compared with that of young cells under oxidative stress. (**A** and **B**) SIRT3 mRNA and total SIRT3 protein expression in young and old hMSCs under oxidative stress. SIRT3 expression is significantly decreased in old cells compared with their younger counterparts. (**C**) The aged hMSCs expressed lower levels of full-length SIRT3 (fl-SIRT3) and the short isoform of SIRT3 (sh-SIRT3) compared with young cells after H_2_O_2_ treatment. (**D**–**H**) mRNA and protein levels of MnSOD and CAT are reduced in older cells treated with H_2_O_2_. (**F** and **I**) MnSOD and CAT enzymatic activity in old hMSCs was significantly lower than that in young ones under the same oxidative stress. (**J**–**L**) Cell survival and apoptosis of young and old groups after H_2_O_2_ treatment. Older cells show increased apoptosis and reduced cell survival under conditions of oxidative stress than do their younger counterparts.

To determine the effect of SIRT3 on cellular ability to defend against detrimental events, we exposed young and old hMSCs to oxidative stress. We observed that after H_2_O_2_ treatment, molecular levels of SIRT3 decreased in both age groups compared with no treatment, but the reduction was more pronounced in old hMSCs (Fig. 5A–C). Both fl-SIRT3 and sh-SIRT3 were reduced after incubation with H_2_O_2_, and both isoforms decreased more significantly in old hMSCs compared to young ones (Fig. 5C). The old hMSCs showed lower antioxidant enzyme expression compared to young ones under oxidative stress (Fig. 5D–H). In terms of enzymatic activity, H_2_O_2_ treatment exacerbated decreased MnSOD and CAT activity in older cells (Fig. 5F and I). Likewise, old hMSCs showed more significant decreased cell survival and increased cell apoptosis after H_2_O_2_ treatment (Fig. 5J–L).

## Discussion

The sirtuins belong to a family of highly conserved genes encoding nicotinamide adenine dinucleotide-dependent deacetylases, which are found in organisms ranging from bacteria to humans 10,34. As a mitochondria-localized deacetylase, SIRT3 has been linked to defence against oxidative stress in a variety of systems 10,13–17,20–25,35. In this study, we used human bone marrow stem cells as an *in vitro* model and human myocardial tissue as *in vivo* model to investigate the effect of ageing on SIRT3 levels and the effect of SIRT3 expression on the cells’ ability to withstand stress.

While most of previous studies were performed on cell lines from humans or other mammals, we chose to focus on cells and tissues from patients, which is more clinically relevant and directly reflects the physiological activity of SIRT3.

Previous studies showed no difference in lifespan between SIRT3-deficient and wild-type mice under normal conditions. However, the ability of SIRT3-deficient mice to withstand oxidative stress is significantly reduced 14,21,36. As these mice display cardiac hypertrophy and fibrosis as ageing progresses and under stressful conditions 14,21,36, we propose that SIRT3 functions as a stress-responsive protein. Our study confirms this assumption in various ways, both in MSCs and in myocardial tissue. As MSCs are highly proliferative and may be useful as cell therapy agents to restore tissue after an injury, we obtained hMSCs from young donors to investigate the functions and mechanisms of SIRT3 in human cells. Oxidative stress was induced in hMSCs by incubation with 1 mM H_2_O_2_ for 1 hr. After severe oxidative stress, SIRT3 levels were down-regulated, revealing an association between SIRT3 and oxidative stress. Significant down-regulation of two leading antioxidant enzymes, MnSOD and CAT, led to decreased antioxidant ability of these cells. This observation is consistent with previous studies that demonstrated that SIRT3 expression can be down-regulated by severe stress 21. We noticed that overexpression of SIRT3 by transfection of a SIRT3 expression construct in cells improves their ability to defend against oxidative damage. The protective effect of increased expression of SIRT3 was also demonstrated by increased cell survival and decreased cells apoptosis in transfected cells compared with control cells.

The function of SIRT3 has been studied extensively in mammalian cells 11–13,15–20,23,25. One of its major molecular functions is to deacetylate the transcription factor Foxo3a, which can then enter the nucleus and drive gene expression of MnSOD and CAT, triggering increased ROS scavenging activity 21,23,24,37. SIRT3 also appears to activate the MnSOD protein directly in response to oxidative stress 38,39. Human SIRT3 has two isoforms: a full-length isoform (fl-SIRT3, 44 kD) and a short one (sh-SIRT3, 28 kD). It is known that the truncated sh-SIRT3 is a mitochondrial-localized active enzyme 11–15,18,19,40,41, but the function of fl-SIRT3 remains controversial 11,15,18–20. Despite the fact both short and long isoforms of SIRT3 were overexpressed in our cell transfection studies, only the levels of sh-SIRT3 were correlated with expression of downstream genes. Increased expression of the SIRT3 downstream targets MnSOD and CAT was also correlated with increased cell survival and decreased apoptosis. To assess SIRT3 functions under conditions of oxidative stress, we compared transfected cells with and without H_2_O_2_ treatment. The results showed that SIRT3 expression along with MnSOD and CAT levels and cell survival were decreased in empty plasmid and pSIRT3-transfected groups under H_2_O_2_ stress, whereas pSIRT3 transfection increased these expression levels significantly compared with the empty plasmid group. We also noticed that, under the same oxidative stress, sh-SIRT3 expression was more correlated with gene expression of MnSOD and CAT as well as cell survival. Given these data, we propose that: (*i*) overexpression of SIRT3 enhances cells’ ability to deal with oxidative stress and reduces stress-mediated cell injury by activating CAT and MnSOD; (*ii*) sh-SIRT3 is likely the primary form functioning to induce oxidative stress tolerance in these cells. Our results indicate for the first time that increased SIRT3 drives CAT/MnSOD expression in hMSCs, and supports an important role for SIRT3 in protection against oxidative stress. In contrast, we did not observe a protective role for the fl-SIRT3 isoform during oxidative stress.

SIRT3 has been studied extensively because it is the only one of the seven sirtuins that has been linked with longevity. SIRT3 has roles in DNA repair, cell cycle progression and lifespan extension which are well-verified in bacteria, but our understanding of its relationship with longevity in humans has been limited to genome association studies while direct molecular evidence has been lacking 29–33. Previous research has demonstrated that a SIRT3 allele containing a variable number of tandem repeats enhancer shows a sex-specific association with longevity and is enriched in males older than 90 years 29. Another study indicates that SIRT3 is a marker associated with longevity in Italian females and German males 30. Other groups also report that SIRT3 and its neighbouring gene PSMD13 are both involved in longevity 31. However, not every study supports a role for SIRT3 in longevity. For instance, Michishita *et al*. found that SIRT3 did not extend the cellular replicative lifespan of normal human fibroblasts 13. Nonetheless, SIRT3 appears to play a critical role in longevity, but the evidence for this role is mainly supported by genomic polymorphism studies rather than research at the molecular level.

A previous study has indicated that SIRT3 was reduced in ageing mouse haematopoietic stem cells (mHSCs), and SIRT3 overexpression in aged mHSCs enhanced their regeneration ability 42. Here for the first time, we have studied the potential association between SIRT3 and longevity/ageing in humans at the molecular level. In our study, young and old hMSCs were collected, cultured and treated with H_2_O_2_. We observed that the ability of ageing cells to defend against severe oxidative injury such as H_2_O_2_ treatment was dramatically reduced compared with younger cells. Furthermore, we found that cell survival was decreased and cell apoptosis increased under this detrimental stress. Thus, our present data unveil a critical role of SIRT3 in maintaining normal antioxidant ability under stress and indicates a potential correlation with cell senescence. It is widely acknowledged that mitochondrial dysfunction is a main instigator leading to ageing, and the mitochondria are the main location of SIRT3 function (though whether SIRT3 is an exclusively mitochondrial protein is still controversial) 8–15,21–23,40,41. Therefore, we suggest that mitochondrial dysfunction in ageing cells results from notably decreased SIRT3 content when severe stress occurs, and the latter contributes to reduced antioxidant ability and eventually to increased cell death. However, how exactly these events proceed needs to be further studied.

Given that the ultimate goal of our studies was to improve health and decrease the clinical challenges of ageing, we also examined young and old human myocardial tissues to explore the status of SIRT3 in ageing hearts. Previous studies in mice had revealed that SIRT3 expression was reduced in old aortic valves compared with young ones, indicating an age-associated SIRT3 reduction 43. In human skeletal muscle, SIRT3 expression was also significantly reduced in elderly individuals when compared to young individuals, and research has shown that mild stimuli like exercise can elevate SIRT3 levels in elderly individuals 44. Although we did not detect any differences in gene and total protein expression of SIRT3 between young and old cardiac tissues (similar to hMSCs), expression of sh-SIRT3 in young myocardial tissue is significantly higher compared with old myocardium. In contrast, we found higher expression of fl-SIRT3 in the aged group compared with young tissues. It is well known that normal mitochondria function contributes to ROS elimination, antioxidant acitivity and prevention of apoptosis 8–11, and that the mitochondria mediate the truncation of fl-SIRT3 to its shorter isoform 11,12. Our data suggest that sh-SIRT3 expression predominates in myocardial tissues, and generation of sh-SIRT3 is impeded in ageing hearts. In addition, CAT and MnSOD gene expression were up-regulated by SIRT3 under oxidative stress in mice 21,23,24,43. Our results showed that CAT gene expression and activity as well as MnSOD activity decreased significantly in the aged tissue group, which was correlated with lower sh-SIRT3, whereas MnSOD expression levels had little difference between two groups. Nonetheless, the decrease in these two antioxidants’ enzymatic activity in the aged group reflects poor antioxidant capacity of older myocardial tissues. In agreement with previous studies, we propose that ageing of cardiac tissues might result from reduction in sh-SIRT3 expression.

### Study limitations and future prospects

As a result of technical difficulties, human MSCs and myocardial tissues from individuals older than 90 years are difficult to obtain, so our aged samples had a mean age of around 60 years. Thus, it is difficult to propose that SIRT3 is directly linked to human longevity. Nonetheless, we have shown that SIRT3 is closely related to antioxidant metabolism, which is important to prevent cellular ageing and maintain the normal functioning of cells. Much remains to be learnt about the effect and mechanisms of action of SIRT3 on cells and further comprehensive studies are needed.
